# The role of neutrophil granule proteins in neuroinflammation and Alzheimer’s disease

**DOI:** 10.1186/s12974-018-1284-4

**Published:** 2018-08-27

**Authors:** Amanda J. Stock, Anne Kasus-Jacobi, H. Anne Pereira

**Affiliations:** 1 The Laboratory of Molecular Gerontology, National Institute on Aging, National Institutes of Health, 251 Bayview Blvd., BRC Rm 06B121, Baltimore, MD 21224 USA; 2 Department of Pharmaceutical Sciences, University of Oklahoma Health Sciences Center, 1110 N. Stonewall Ave., CPB 255, Oklahoma City, OK 73117 USA; 3 Oklahoma Center for Neuroscience, University of Oklahoma Health Sciences Center, 1110 N. Stonewall Ave., CPB 255, Oklahoma City, OK 73117 USA; 4 Department of Cell Biology, University of Oklahoma Health Sciences Center, 1105 N. Stonewall, Robert M. Bird Library, Rm 258, Oklahoma City, OK 73117 USA; 5 Department of Pathology, University of Oklahoma Health Sciences Center, 1105 N. Stonewall, Robert M. Bird Library, Rm 258, Oklahoma City, OK 73117 USA

**Keywords:** Neutrophils, CAP37, Neutrophil elastase, Cathepsin G, Amyloid beta, RAGE, Neuroinflammation, Alzheimer’s disease

## Abstract

Neutrophils are the innate immune system’s first line of defense. Neutrophils play a critical role in protecting the host against infectious pathogens, resolving sterile injuries, and mediating inflammatory responses. The granules of neutrophils and their constituent proteins are central to these functions. Although neutrophils may exert a protective role upon acute inflammatory conditions or insults, continued activity of neutrophils in chronic inflammatory diseases can contribute to tissue damage. Neutrophil granule proteins are involved in a number of chronic inflammatory conditions and diseases. However, the functions of these proteins in neuroinflammation and chronic neuroinflammatory diseases, including Alzheimer’s disease (AD), remain to be elucidated. In this review, we discuss recent findings from our lab and others that suggest possible functions for neutrophils and the neutrophil granule proteins, CAP37, neutrophil elastase, and cathepsin G, in neuroinflammation, with an emphasis on AD. These findings reveal that neutrophil granule proteins may exert both neuroprotective and neurotoxic effects. Further research should determine whether neutrophil granule proteins are valid targets for therapeutic interventions in chronic neuroinflammatory diseases.

## Background

Neutrophils are the most abundant leukocytes in the human circulatory system [1]. Although the most well-known function of neutrophils is defending the host against infectious pathogens, they also facilitate the repair of sterile wounds and mediate inflammation resulting from infectious and sterile injuries [2]. The production and differentiation of neutrophils occur in the bone marrow, where they are formed at a rate of ~ 16 × 10^10^ cells/day in humans [3]. Neutrophils are unique among other immune cells due to their short half-lives of 6–8 h, rapid response, and ability to capture microbes with neutrophil extracellular traps (NETs), which are protruding structures consisting of decondensed chromatin and antimicrobial/granular proteins that allow the neutrophils to eliminate extracellular pathogens [1–3]. They are able to rapidly migrate towards regions of injury or infection, phagocytose pathogens and debris, and release reactive oxygen species, cytokines, chemokines, proteases, and antimicrobial proteins and peptides that help kill bacteria and regulate inflammation [2]. While neutrophil antimicrobial and inflammatory functions are generally considered beneficial, prolonged activation of neutrophils can also contribute to tissue damage [1]. It is known that neutrophils play a role in a number of chronic inflammatory conditions and diseases, including cystic fibrosis [4], chronic obstructive pulmonary disease [5], atherosclerosis [1], and rheumatoid arthritis [6]. However, the involvement of peripheral neutrophils and neutrophils in the brain in chronic inflammatory neurodegenerative diseases, such as Alzheimer’s disease (AD), remains to be elucidated.

In this review, we focus on three neutrophil granule proteins: the cationic antimicrobial protein of 37 kDa (CAP37), neutrophil elastase, and cathepsin G. We discuss their expression by non-neutrophil cells in the brain and in the periphery, and the functions of these proteins that could be either protective or harmful under normal physiological or neuroinflammatory conditions. Findings from our lab [7–10] that suggest a potential role for these neutrophil proteins in neuroinflammation and AD will be reviewed.

## Alzheimer’s disease

AD is the most common cause of dementia and the sixth leading cause of death in the USA [11]. The two major pathological hallmarks of AD are the presence of senile plaques containing amyloid beta (Aβ) peptides and tau protein-containing neurofibrillary tangles, derived from hyperphosphorylation of the microtubule-associated protein tau [12]. The etiology of late-onset AD is unknown, but is believed to be multifactorial [13]. Some pathological events hypothesized to contribute include excitotoxicity due to excessive glutamate levels, decreased acetylcholine neurotransmission, oxidative stress, disruption of the blood-brain barrier (BBB), decreased glucose metabolism, and vascular dysfunction, which can collectively contribute to memory impairment [14]. Neuroinflammation is another major pathological feature associated with AD [14], which we will discuss in this review. The involvement of microglia and astrocytes in neuroinflammation associated with AD is well established [14]. Additionally, peripheral immune cells, including monocytes and T cells, have been found to traverse the BBB [14, 15], and researchers have been investigating the effects of these immune cells in the brains of AD patients for a number of years [16–20]. In contrast, the role of neutrophils in the brains of AD patients has been under-appreciated and under-studied. Our recent findings demonstrate that specific neutrophil proteins may regulate neuroinflammation associated with AD [9, 10]. These findings emphasize the importance of investigating neutrophils in AD.

## Neutrophils in Alzheimer’s disease

Modest research has been performed to investigate the role of neutrophils in AD. A report by Scali et al. [21] demonstrated that the CD11b integrin was upregulated in peripheral blood neutrophils of patients with AD. Increased neutrophil expression of CD11b, which supports neutrophil adhesion and migration, positively correlated with disease severity. A different study by Vitte et al. [22] revealed increased levels of reactive oxygen species in peripheral blood neutrophils from patients with AD compared with controls. These findings suggest that neutrophils may exist in a more activated state during AD. In more recent studies, the use of two-photon laser imaging on mouse models of AD revealed that neutrophils were traversing the BBB into the brain. Baik et al. [23] showed live imaging of neutrophils entering the brain parenchyma in 5XFAD mice, a mouse model of AD. This migration was not seen in wild-type control mice. Importantly, the neutrophils that entered the parenchyma remained motile and accumulated around amyloid beta (Aβ) plaques in the AD mice. These findings were corroborated in a report by Zenaro et al. [24]. This report demonstrated increased neutrophils in the brain parenchyma of 5XFAD mice as well as 3xTg-AD mice, another mouse model of AD. Two-photon laser imaging confirmed the extravasation of neutrophils into the brain parenchyma of the 5XFAD mice. In this study, Aβ increased the affinity state of lymphocyte function-associated antigen-1 (LFA-1), a leukocyte integrin expressed on neutrophils that binds to adhesion molecules like ICAM-1. Since Aβ enhanced the affinity state of LFA-1, the authors concluded that the increased neutrophil adhesion was likely dependent on the effects of Aβ on LFA-1. Binding of neutrophils to ICAM-1 on brain endothelial cells could lead to neutrophil transmigration into the brain parenchyma. In AD mice that were LFA-1 deficient, neutrophils did not infiltrate the brain parenchyma, indicating that neutrophil infiltration was facilitated by LFA-1. Depletion of neutrophils or LFA-1 improved cognitive function, decreased microgliosis, and decreased the levels of Aβ_1–42_ in the brain homogenates of 3xTg-AD mice. Furthermore, neutrophil depletion decreased the levels of phosphorylated tau (phospho-Ser202 and phospho-Thr205) in the AD mice. In addition, this study demonstrated higher numbers of neutrophils in the cerebral blood vessels and brain parenchyma of human AD patients compared with age-matched controls. Overall, these findings suggest the possibility that neutrophils could contribute to AD pathology.

## Neutrophil granule proteins

Neutrophils contain four types of granules, all of which are formed during neutrophil differentiation. The four types of granules are the azurophil, specific, gelatinase, and secretory granules [25]. Since the granules are formed during neutrophil differentiation, their constituent proteins are “pre-packaged,” and readily available to be released to participate in various functions in the host’s response to infection or inflammation [26]. When neutrophils are activated, the contents of the secretory granules are the quickest to undergo exocytosis (during the early stage of neutrophil activation), followed by the release of the contents of the gelatinase granules, and specific granules. Azurophil granules experience limited exocytosis, which occurs during the late stage of neutrophil activation. The contents of azurophil granules function primarily within the phagolysosomes [27]. Some of the major proteins found in azurophil granules are antibacterial proteins like myeloperoxidase and CAP37 [28], also known as azurocidin [29] or heparin binding protein [30]. Proteases that degrade extracellular matrix proteins, facilitate immune receptor activation and inactivation, and assist in digestion and clearance of pathogens are also present in azurophil granules [28]. These proteins include neutrophil elastase, cathepsin G, and proteinase-3. The specific granules harbor the antibacterial proteins lactoferrin, neutrophil gelatinase-associated lipocalin, cathelicidin, and lysozyme [28]. Proteases, such as collagenase, are also found in specific granules. Major constituent proteins of gelatinase granules include the antibacterial protein, lysozyme, and the proteases gelatinase (also known as matrix metalloproteinase 9, MMP9) and leukolysin (MMP25). Secretory granules are rich in transmembrane receptors (e.g., tumor necrosis factor receptors and interferon-α receptors) that integrate into the plasma membrane of neutrophils as exocytosis takes place. The secretory granules do not contain many antibacterial proteins or proteases [28]. However, the antimicrobial protein CAP37, unlike most other azurophil granule proteins, is also found in secretory granules. Therefore, CAP37 is secreted during both the early and late stages of neutrophil activation [29].

## CAP37, neutrophil elastase, and cathepsin G

We focus on the neuroinflammatory role of CAP37 in this review since our lab has demonstrated effects of CAP37 on microglial functions [8]. In addition, we previously investigated the expression of CAP37 in AD, and demonstrated an upregulation of CAP37 in patients with AD [7, 9]. We will discuss the role of two other neutrophil proteins: neutrophil elastase and cathepsin G. CAP37 shares approximately 45% and 32% sequence identity with neutrophil elastase and cathepsin G, respectively [31]. All three neutrophil proteins share a similar three-dimensional structure, with two homologous β-barrels, formed by six anti-parallel β strands and a C-terminal α-helix [32, 33]. Furthermore, a correlation analysis revealed that gene expression of neutrophil elastase and cathepsin G positively correlated with CAP37 expression [10]. Neutrophil elastase and cathepsin G, which are serine proteases, cleave various extracellular matrix proteins and share many of the same substrates [34]. Although CAP37 was previously believed to lack serine protease activity [35, 36], this theory has become controversial. Recent reports have demonstrated that insulin-like growth factor binding proteins-1, -2, and -4 can be cleaved by CAP37 [37, 38]. In addition, our lab showed cleavage of the Alzheimer’s-associated peptide, Aβ, by CAP37 [10], which will be discussed in a later section of this review.

**Fig. 1:**
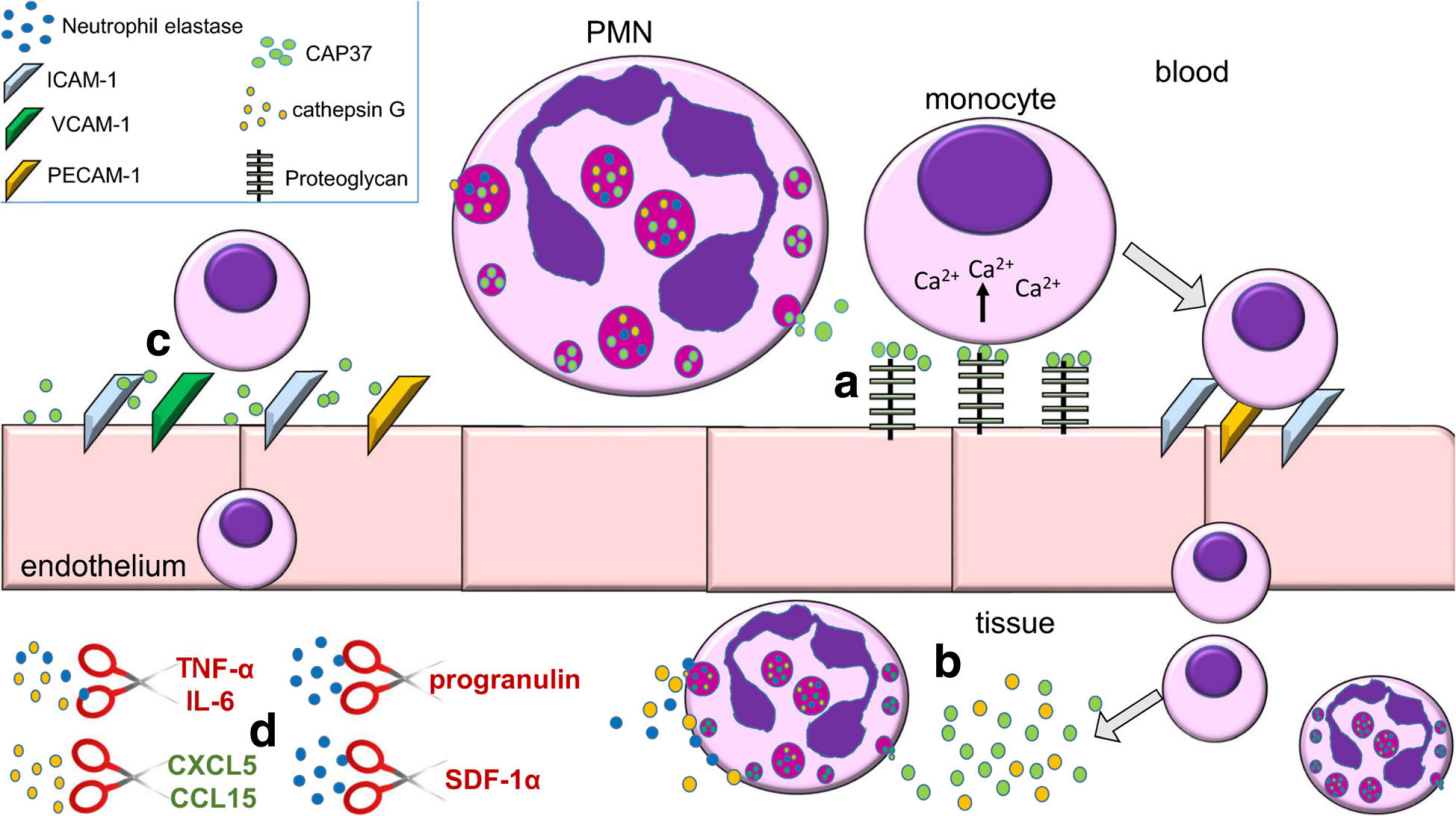
Effects of the neutrophil (PMN) proteins CAP37, neutrophil elastase, and cathepsin G on inflammation and host defense. **a** CAP37 released from secretory or azurophil granules of PMN can accumulate on proteoglycans on endothelial cells. CAP37 on proteoglycans activates monocytes by increasing intracellular calcium mobilization which leads to increased monocyte adhesion to endothelial cells. **b** CAP37 and cathepsin G released from neutrophils within the injured/infected tissues are chemotactic for monocytes, causing more monocytes to extravasate into the tissue. **c** CAP37 increases the expression of adhesion proteins (ICAM-1, VCAM-1, PECAM-1) on endothelial cells, leading to the attachment and extravasation of more monocytes and PMNs into the tissue. **d** Secreted neutrophil elastase and cathepsin G cleave various cytokines and chemokines leading to their activation (green) or inactivation (red). Neutrophil elastase cleaves progranulin, an anti-inflammatory protein, and prevents its anti-inflammatory functions. Neutrophil elastase also cleaves and inactivates the chemokine SDF-1α. Both neutrophil elastase and cathepsin G cleave and inactivate the pro-inflammatory cytokines TNF-α and IL-6. Cathepsin G cleaves and activates the chemokines CXCL5 and CCL15 leading to recruitment of more immune cells

In neutrophils, the primary functions of CAP37, neutrophil elastase, and cathepsin G are to defend the host against microbial pathogens and to mediate inflammation [33, 39]. Upon activation, neutrophils attach to vascular endothelial cells to prepare for extravasation into infected and injured tissue. CAP37 is released from the azurophil and secretory granules of neutrophils, and adheres to the negatively charged proteoglycans on the surface of the endothelium **(**Fig. 1**)** [36]. While attached to the proteoglycans, CAP37 recruits and activates monocytes by increasing their intracellular Ca^2+^ [40, 41]. In addition, CAP37 has been shown to increase the expression of adhesion molecules, including ICAM-1, platelet endothelial cell adhesion molecule 1 (PECAM-1), vascular cell adhesion protein 1 (VCAM-1), and E-selectin, on endothelial cells [42, 43]. This further promotes the adherence and extravasation of additional neutrophils and monocytes. Once neutrophils extravasate into infected and injured tissues, they phagocytose microorganisms and debris. Upon ingestion, the microorganisms are taken up into phagosomes. Azurophil granules harboring CAP37, neutrophil elastase, and cathepsin G fuse with the phagosomes to form phagolysosomes and implement killing through a non-oxidative mechanism [33].

CAP37 has biological effects on various mammalian cells including endothelial cells, monocytes, and macrophages [29, 44]. For example, rearrangement of the endothelial cell cytoskeleton and subsequent endothelial cell contraction induced by CAP37 lead to increased vascular permeability [45]. Our lab previously demonstrated the effects of CAP37 on smooth muscle cells, including induction of vascular smooth muscle cell migration and proliferation and increased ICAM-1 expression [46]. Importantly, CAP37 also recruits other immune cells to sites of injury and infection through its chemotactic activity for monocytes [47] and macrophages [36]. CAP37 induces the release of the cytokines TNF-α and interferon gamma (IFN-γ) from macrophages and enhances the phagocytosis of bacteria by macrophages [48].

Although neutrophil elastase and cathepsin G are not stored and released from secretory granules, they can be transported to the plasma membrane and released from azurophil granules to exert inflammatory activities in the extracellular space **(**Fig. 1**)** [25]. In the extracellular environment, neutrophil elastase and cathepsin G cleave chemokines and cytokines, leading to their activation or inactivation. Like CAP37, cathepsin G is chemotactic for monocytes. However, the chemotactic activity of cathepsin G has been determined to be dependent on its enzymatic activity [25].

CAP37, neutrophil elastase, and cathepsin G are not restricted to neutrophils, as they have been identified in other mammalian cells. Neutrophil elastase and cathepsin G are expressed in a low percentage of monocytes. CAP37 was expressed in endothelial cells and smooth muscle cells within areas of atherosclerotic lesions [46, 49]. Expression of CAP37 is induced in the corneal epithelium, limbus, ciliary epithelium, ciliary vascular endothelium, and stromal fibroblasts in a rabbit model of bacterial keratitis [50]. Increased levels of CAP37 in plasma have been described in patients with sepsis [51].

## CAP37, neutrophil elastase, and cathepsin G in neuroinflammation

The roles of CAP37, neutrophil elastase, and cathepsin G in inflammation are well documented [25, 29, 33, 49]. However, the inflammatory roles of these proteins in the central nervous system (CNS) are not well known. These proteins are expressed in the CNS upon certain inflammatory insults or diseases [52–54]. The expression of neutrophil elastase and cathepsin G has been demonstrated in microglial cells [55, 56] and in the cerebrospinal fluid [52, 53]. In a study by Linder et al., [52] significantly higher levels of CAP37 were found in the cerebrospinal fluid (CSF) of patients with acute bacterial meningitis than in patients with viral meningitis, viral encephalitis, neuroborreliosis, and normal controls. Another study revealed that neutrophil elastase activity was significantly higher in the CSF of patients with purulent meningitis than in patients with aseptic meningitis [53].

Previous literature has indicated that neutrophil elastase and/or cathepsin G may be involved in the pathology associated with traumatic brain injury, neuromyelitis optica, and ischemic stroke [54–56]. In a study by Semple et al., [54] mice were inflicted with a brain injury at postnatal day 21 using the controlled cortical impact model of traumatic brain injury to mimic injury to a young human brain (approximately 2 years old). This study demonstrated that mice deficient in neutrophil elastase exhibited less vasogenic edema and cell death, and had improved spatial memory retention compared with wild-type mice, indicating that neutrophil elastase may contribute to brain damage and pathology. Neuromyelitis optica is an inflammatory disease in which neuron demyelination occurs in the optic nerve and spinal cord. In a study investigating the effects of neutrophils in a mouse model of neuromyelitis optica, intracerebral injection of inhibitors of neutrophil elastase and cathepsin G, as well as intraperitoneal injection of neutrophil elastase inhibitor alone, reduced neuromyelitis optica brain lesions [57]. Inhibition of cathepsin G has also been found to increase cerebral blood flow and reduce infarct volume and neurobehavioral deficits in a mouse model of ischemic stroke [58].

CAP37 may play a role in neuroinflammation by modulating the functions of microglial cells. The resident macrophages of the brain, known as microglia, are the predominant modulators of neuroinflammation [59]. In their resting state, microglia exhibit a stretched or ramified morphology while scanning the microenvironment for potential pathogens. When microglia are activated by pathogen-associated molecular patterns or damage-associated molecular patterns, they display an amoeboid morphology [60–63]. Chemotactic receptors expressed on activated microglia allow them to sense ATP or chemokines released from cells surrounding the site of injury/infection and to migrate towards this site by following ATP or chemotactic gradients [64, 65]. Activated microglia express increased levels of complement receptors and major histocompatibility molecules, and they release growth factors, chemokines, pro-inflammatory cytokines, and pro-oxidant molecules, including TNF-α, IL-1β, superoxides, and nitric oxide [64, 66]. Additionally, active microglial cells phagocytose harmful debris and pathogens that could damage surrounding brain cells [61].

During certain neurodegenerative diseases, such as AD, microglial functions become dysregulated, and prolonged neuroinflammation likely contributes to neurotoxicity [65, 66]. Increased numbers of activated microglial cells [67, 68] and higher levels of pro-inflammatory cytokines are present in the brains of patients with AD [69, 70]. Interestingly, a rare mutation on the triggering receptor expressed on myeloid cells 2 (TREM2), a gene that facilitates microglial phagocytosis, increases the risks of neurodegenerative diseases, including AD [71]. Thus, dysregulated phagocytic activity of microglia likely contributes to neuronal death and dysfunction in AD [72].

Our lab found that CAP37 activates microglial cells [8]. N9 mouse microglial cells treated with CAP37 displayed an amoeboid (active) morphology, released higher levels of the pro-inflammatory cytokines, TNF-α and interleukin-1 beta (IL-1β), and demonstrated increased expression of the chemokines RANTES and fractalkine [8]. In vitro assays demonstrated that CAP37 induced chemotaxis of the microglial cells and enhanced their phagocytic activity towards serum-opsonized zymosan A particles.

The collective findings on the involvement of CAP37, neutrophil elastase, and cathepsin G in neuroinflammatory processes and diseases of the CNS indicate that these proteins may be important factors in neuroinflammation. Further investigation is needed to determine the exact roles of these proteins in chronic neuroinflammation-mediated neurodegenerative diseases, such as AD.

## CAP37 and Alzheimer’s disease

Published data from our laboratory demonstrate the increased expression of CAP37 in brains of patients with AD [7, 9]. Immunohistochemistry (IHC) performed on hippocampal tissues revealed the presence of CAP37 in hippocampal endothelial cells of patients with AD [7]. CAP37 expression was not observed in the brain endothelial cells of normal controls or patients with Parkinson’s disease, Binswanger disease, Progressive supranuclear palsy, Candida microabscesses, or frontotemporal dementia (FTD, Pick’s disease). These findings indicated that CAP37 expression in the hippocampal vasculature may be induced in response to disease-specific pathological factors. In line with this notion, the same study demonstrated that CAP37 expression was induced by amyloid beta 1–40 (Aβ_1–40_) in rat brain endothelial cells. These findings suggest that Aβ may be a causative factor of the expression of CAP37 observed in patients with AD. Our more recent report [9], which was designed to look for expression of non-neutrophilic CAP37 in brain regions other than the hippocampal vasculature in AD patients, expands on these previous findings. In this new study, immunohistochemistry, western blotting, and quantitative reverse transcriptase polymerase chain reaction (qRT-PCR) were performed on various brain regions from patients with Alzheimer’s disease and normal controls.

Immunohistochemistry with a monoclonal antibody that was developed in our lab demonstrated CAP37 positive staining in pyramidal neurons of the temporal and parietal neocortices. CAP37 expression was also revealed in the CA3 and CA4 pyramidal neurons of the hippocampus. Positive staining for CAP37 was observed in more pyramidal neurons in the temporal and parietal neocortices of patients with AD than in age-matched controls. The expression of CAP37 in neurons was confirmed by qRT-PCR analysis. In addition, qRT-PCR revealed a significant 8- and 12- fold increase in the levels of AZU1 (CAP37) mRNA in the temporal lobe and frontal lobes of patients with AD, respectively, compared with normal controls. The transcript levels of ELANE (neutrophil elastase) and CTSG (cathepsin G) were not significantly higher in patients with AD, suggesting that CAP37 may be specifically induced in brain cells. However, it is not known whether the proteolytic activity of neutrophil elastase and cathepsin G are altered in AD. The temporal, parietal, and frontal lobes where CAP37 was induced are brain regions that are highly impacted by AD pathology [73]. Interestingly, the occipital lobe is one of the least impacted brain regions in AD, [73] and we did not observe a significant increase of CAP37 in the occipital lobes of patients with AD. These results, which demonstrated increased CAP37 in the brain regions that undergo the greatest atrophy during AD, further support our hypothesis that CAP37 is associated with the pathogenesis of AD.

Since we found that Aβ_1–40_ induced the expression of CAP37 in brain endothelial cells [7], we predicted that Aβ_1–40_ might also be able to induce the expression of CAP37 in neurons. This was investigated by performing immunocytochemistry on primary human cortical neurons treated with Aβ_1–40_ overnight [9]. Neurons were also treated with the pro-inflammatory cytokine TNF-α, which is increased in the brains of patients with AD. Results demonstrated that Aβ_1–40_ and TNF-α induced the expression of CAP37 within the cell bodies of the cortical neurons. The distinct staining of CAP37 was not observed in vehicle-control treated neurons or in response to the reverse/inactive Aβ peptide (Aβ_40–1_). Therefore, expression of CAP37 in the brain may occur in response to the elevated levels of Aβ and pro-inflammatory cytokines that are present in AD. We posit that CAP37 may respond to and mediate neuroinflammation in AD.

## CAP37 and other neurodegenerative diseases

Since we revealed that CAP37 was upregulated in neurons of patients with AD, we investigated whether CAP37 was present in brain cells of other patients with immune-mediated neurodegenerative diseases. CAP37 expression in other immune-mediated neurodegenerative diseases was determined by performing IHC with anti-CAP37 or mouse isotype control antibody. IHC was performed on temporal and parietal neocortices from a patient with frontotemporal dementia (FTD), a patient with vascular dementia (VaD), a patient with AD + diffuse Lewy body dementia (AD + DLBD), and corresponding age-matched controls. These findings are summarized in Table 1. Although we observed moderate to strong staining in the temporal neocortical neurons of patients with FTD and AD + DLBD, we noted minimal positive staining for CAP37 in neurons from the same regions of VaD patients. Similar results were observed upon analyzing parietal neocortices stained for CAP37. However, the intensity of CAP37 positivity in the neocortical neurons was lower in the parietal lobe than in the temporal lobe of the AD + DLBD patient. These results suggest that the level of CAP37 expression in neurons of the brain is heterogeneous and may depend on the neural environment and the presence of disease-specific pathological constituents. We are cognizant that our analysis of CAP37 expression in one patient with each disease may not give an accurate representation of the disease specificity of CAP37 in general. More patients with these diseases should be analyzed for CAP37 expression in neurons before any solid conclusions are made.

**Table 1:** CAP37 positive staining in neurons of patients with neurodegenerative disease

	FTD	VaD	AD + DLBD
Temporal neocortex	Moderate/strong	Minimal	Moderate/strong
Parietal neocortex	Moderate/strong	Minimal	Minimal/moderate

## Receptors for CAP37, neutrophil elastase, and cathepsin G

Identifying the receptor(s) for CAP37, neutrophil elastase, and cathepsin G would provide important knowledge on the functions of these proteins in neuroinflammation and AD. Although specific receptors for these neutrophil proteins have not yet been confirmed, reports have revealed different receptors predicted to be activated or inactivated in response to these proteins. The β_2_ integrins are receptor subunits expressed on all leukocytes and are critical for cell-cell adhesion. In a study by Soehnlein et al., [40] treatment with CAP37 induced intracellular Ca^2+^ mobilization in the MM6 monocytic cell line and increased monocyte adhesion to activated endothelial cells. Another report by Påhlman et al. demonstrated that the CAP37-induced Ca^2+^ mobilization was inhibited with an antibody for β_2_ integrins, indicating that CAP37 may augment monocyte activation by signaling through receptors consisting of β_2_ integrins [74]. A report by Zen et al. [75] demonstrated that neutrophil elastase, cathepsin G, and proteinase-3 all cleaved the extracellular domain of CD11b integrin, which was predicted to allow for the detachment of neutrophils from CD11b integrins and the subsequent transmigration of neutrophils across the endothelium. These studies indicate that CAP37, neutrophil elastase, and cathepsin G may each have important roles in mediating leukocyte functions through integrins.

Various G-protein-coupled receptors are modulated by CAP37, neutrophil elastase, and cathepsin G. CAP37-induced chemotaxis of corneal epithelial cells, and cathepsin G-induced chemotaxis of monocytes are significantly decreased when cells are treated with pertussis toxin [76, 77]. Since pertussis toxin is known to inhibit G-protein-coupled receptor signaling, this indicates that CAP37 and cathepsin G may induce their chemotactic effects by signaling through a G-protein-coupled receptor that remains to be identified. An article by Sun et al. [78] revealed that cathepsin G-induced monocyte chemotaxis was inhibited with an antibody for formyl peptide receptor and the formyl peptide receptor antagonist, cyclosporine H. Another study demonstrated that cathepsin G cleaved formyl peptide receptor agonists allowing for their extracellular release and subsequent induction of formyl peptide receptor-dependent chemokine (C-X-C motif) ligand 2 (CXCL2) release [79]. These studies indicate that cathepsin G may mediate monocyte functions such as chemotaxis through the G-protein-coupled formyl peptide receptors.

Protease-activated receptors (PARs) are a family of G-protein-coupled receptors that are cleaved by neutrophil elastase and cathepsin G. To date, four different PARs have been identified [33]. These receptors are expressed primarily on platelets and endothelial cells and are involved in functions such as coagulation, vascular tone, and inflammation [80]. Cathepsin G can activate PAR-4 on platelets, leading to platelet aggregation [81]. On the other hand, PAR-1, the receptor for thrombin, is cleaved by neutrophil elastase and cathepsin G, leading to its inactivation on endothelial cells and platelets [33, 82].

Although the majority of receptors modulated by neutrophil elastase and cathepsin G are cleaved directly by these proteases, leading to their activation or inactivation, whether the effects of CAP37 on receptors are due to enzymatic activity has yet to be determined. CAP37-induced chemotactic effects appear to be dependent upon G-protein-coupled receptor signaling, and CAP37 signaling through β_2_ integrins may be important for monocyte adhesion to endothelial cells. The specific G-protein-coupled receptor and β_2_ integrin underlying these effects must still be identified. It is also uncertain whether either of these receptors are involved in the induced phagocytic activity and upregulation of chemokines, cytokines, and/or adhesion molecules that occur in specific cells in response to CAP37. It is possible that one or more other receptors are involved in these functions of CAP37.

## RAGE and amyloid beta in neuroinflammation and Alzheimer’s disease

A recent report from our lab [10] demonstrated a positive correlation between expression of CAP37 and ligands for the receptor for advanced glycation end-products (RAGE). This led us to investigate whether CAP37 could also serve as a ligand for RAGE. Binding of CAP37, neutrophil elastase, and cathepsin G to RAGE was observed, but whether these neutrophil proteins act as agonists or antagonists of RAGE remains unknown. RAGE is a pattern recognition receptor (PRR) and a member of the immunoglobulin superfamily that recognizes a variety of ligands, including the advanced glycation end-products (AGEs) that are well known for their role in diabetes and atherosclerosis; Aβ, found in the senile plaques of AD brains; inflammatory mediators, such as members of the S100/calgranulin family, high mobility group box 1 (HMGB-1); and the adhesion molecule Mac-1 [83, 84]. RAGE is expressed on neurons, microglia, astrocytes, and endothelial cells in the brain [85–87]. It is expressed at low levels, but can be upregulated during inflammatory conditions or disease states [88]. In patients with AD, RAGE expression is increased in the vasculature, hippocampus, and frontal lobe [85, 89].

**Fig. 2:**
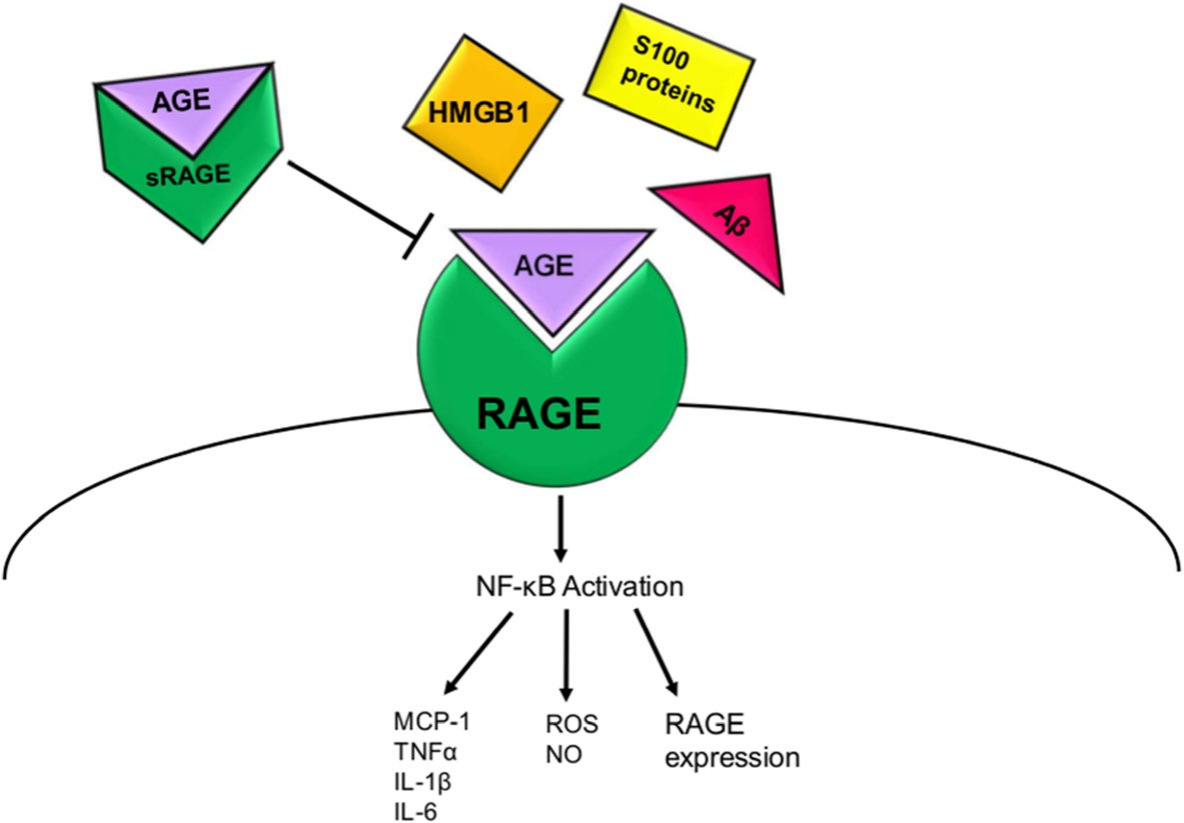
Ligands of RAGE activate NF-κB. Ligands of RAGE include amyloid beta (Aβ), high-mobility group box-1 (HMGB1), advanced glycation end products (AGEs), and S100 proteins. Binding of ligands to full-length RAGE located on the cell membrane induces a cell signaling cascade that leads to activation of the transcription factor NF-κB. Activation of NF-κB stimulates the production of pro-inflammatory cytokines including TNF-α, IL-1β, and IL-6, pro-oxidants including reactive oxygen species (ROS) and nitric oxide (NO), as well as RAGE itself. Soluble RAGE acts as a decoy receptor to bind other RAGE ligands and prevent them from activating cell signaling through full-length RAGE

Binding of agonist ligands to full-length RAGE is known to activate a signaling cascade that leads to the activation of the transcription factor NF-κB, which augments the transcription of various pro-inflammatory cytokines, chemokines, and pro-oxidant genes [90] **(**Fig. 2**)**. One of the unique aspects of RAGE is its perpetual feed-forward signaling following activation. It has previously been demonstrated that ligand binding to RAGE increases RAGE expression to further exacerbate the initial response [83]. Soluble forms of RAGE that only contain the extracellular domain of RAGE exist. Soluble RAGE (sRAGE) binds to ligands, but does not activate cell responses, and therefore acts as a decoy receptor to prevent RAGE signaling.

Interestingly, in addition to their interaction with RAGE, we found a direct interaction between neutrophil proteins (CAP37, neutrophil elastase, and cathepsin G) and Aβ [10]. It is known that the Aβ peptides are the main components of senile plaques in AD. Aβ peptides are derived from the amyloid precursor protein (APP) that is expressed on the cell membranes of neurons [12]. The enzymes α- , β- , and γ-secretase cleave APP, leading to the generation of different protein fragments. APP is processed by these enzymes through a non-amyloidogenic or an amyloidogenic pathway. The two most common Aβ peptides that are formed through the amyloidogenic pathway are Aβ_1–40_ and Aβ_1–42_. Approximately 90% of Aβ peptide formed is Aβ_1–40_, and 10% is Aβ_1–42_ [91]. Aβ peptides can aggregate in the extracellular space around neurons to form soluble oligomers, protofibrils, fibrils, and insoluble amyloid plaques, which can each cause different levels of neurotoxicity [12]. Soluble aggregates of Aβ are neurotoxic; since Aβ_1–42_ aggregates more readily than Aβ_1–40_, it is considered to be the more toxic of the two peptides. In addition, elevated levels of Aβ_1–42_ relative to less toxic Aβ_1–40_ appear to be correlated with AD [92–94].

Mutations in the presenilin genes, which constitute the catalytic domain of γ-secretase, and in the APP gene are the main causes of early-onset familial Alzheimer’s disease [95]. These mutations lead to the increased production and accumulation of Aβ. The positive correlation between genetic mutations that increase Aβ deposition and AD supports the notion that Aβ accumulation promotes disease progression. The hypothesis that Aβ leads to a cascade of pathological events that ultimately causes neurotoxicity and cognitive decline in AD is known as the amyloid cascade hypothesis. This hypothesis was proposed by Hardy et al. in 1992 [95], and it has dominated the field of AD research for the last 25 years. Unfortunately, a number of candidate therapies for AD designed to target Aβ have reached clinical trials but have failed due to lack of efficacy or severe side effects. These studies bring into question whether targeting Aβ is a practicable approach for treating AD patients. However, it is worth noting that these trials were carried out after Aβ accumulation. It is possible that therapeutics targeting Aβ would be more effective if administered before the Aβ accumulation could cause neurotoxicity.

Several reports have indicated that RAGE signaling contributes to the neuronal dysfunction and cognitive deficits that occur in response to Aβ [89, 96, 97]. Activation of RAGE by Aβ oligomers and fibrils leads to toxic effects, such as breakdown of the blood-brain barrier, neuroinflammation, and oxidative stress, all hallmarks of AD associated with cognitive decline [98]. In contrast, activation of RAGE by Aβ monomers seems to be non-toxic and induces neuronal differentiation [98]. RAGE expression is increased in neurons, microglia, and endothelial cells in the brains of patients with AD, leading to heightened Aβ-RAGE signaling [85, 89].

Aβ can evoke various functions in different cells of the brain by signaling through RAGE. In astrocytes and cerebral endothelial cells, the activation of RAGE by Aβ induces oxidative stress by increasing the production of reactive oxygen species [99, 100]. Aβ binding to RAGE on neurons induces the release of macrophage colony stimulating factor (M-CSF), which then activates microglial cells [101]. Various studies have demonstrated RAGE involvement in microglial functions. Ligand activation of RAGE has been shown to induce the release of the pro-inflammatory cytokines TNF-α, IL-1β, and IL-6, as well as chemokines, including macrophage inflammatory protein 1-α (MIP-1α), chemokine ligand 5 (CCL5), and stromal cell-derived factor 1 (SDF-1), from microglia [88].

In a study by Arancio et al., [102] mice expressing mutant amyloid precursor protein (mAPP) were used as models of AD pathology. To determine the effect of the Aβ-RAGE interaction on neuronal function and cognition in vivo, double transgenic (Tg) mice expressing mAPP and overexpressing RAGE in neurons were generated (Tg mAPP/RAGE) [102]. In this study, Tg mAPP/RAGE mice had significantly higher numbers of active microglia and astrocytes, showed greater behavioral cognitive deficits, had reduced long term potentiation, and had diminished acetylcholinesterase-positive neurites compared with the three control groups, (Tg mAPP, Tg RAGE, and non-transgenic [non-Tg] littermates). These findings suggest that the presence of high levels of RAGE in the brains of patients with AD could exacerbate Aβ-induced inflammation, neuronal dysfunction, and cognitive impairment.

**Fig. 3:**
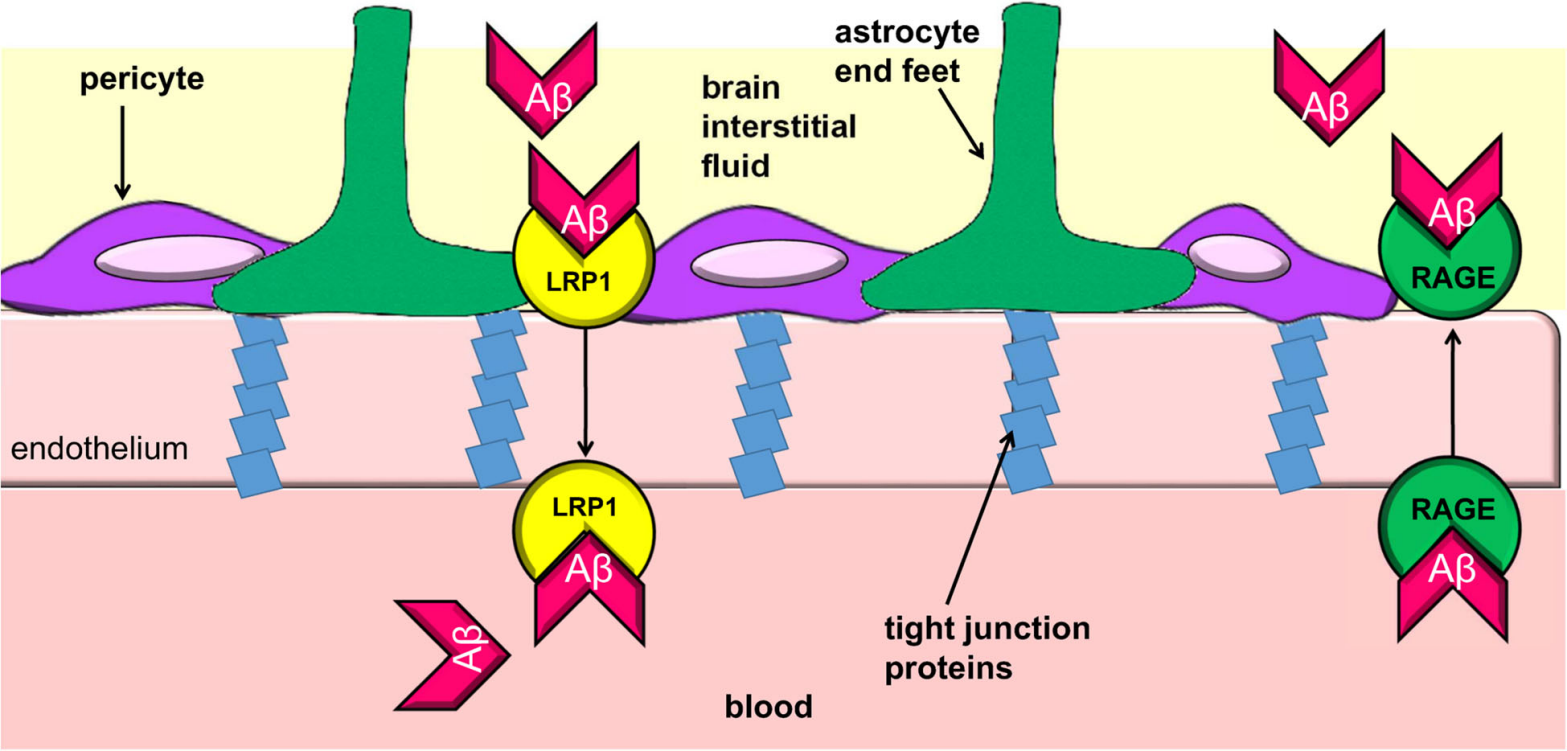
Amyloid beta crosses the blood-brain barrier by receptor mediated transport. The blood-brain barrier (BBB) is composed of endothelial cells connected by tight junction proteins. Pericytes and astrocyte end-feet around the endothelial cells support BBB integrity. Aβ can enter and exit the brain by receptor-mediated transcytosis. RAGE is the receptor transporting Aβ from the blood into the brain and low-density lipoprotein receptor-related protein 1(LRP-1) is the receptor transporting Aβ from the brain to the blood. An increase in the expression of RAGE and decrease in the expression of LRP-1 supports the increased accumulation of Aβ in AD

At the BBB, RAGE is the main receptor that transports Aβ from the blood into the brain, while low density lipoprotein receptor-related protein 1 (LRP1) is the predominant receptor that facilitates Aβ transport from the brain into the circulation [103, 104] (Fig. 3). Importantly, expression of LRP1 is decreased, and the expression of RAGE is increased in patients with AD, these expression levels may support brain accumulation of Aβ [104]. Studies have also demonstrated that Aβ may contribute to BBB disruption [105–107]. The integrity of the BBB is supported by pericytes, specialized vascular cells that wrap around endothelial cells, and astrocyte end feet [108]. Endothelial cells and the tight junctions that connect them constitute the BBB [103] **(**Fig. 3**)**. A previous report by Wan et al. [107] demonstrated that treatment of a brain endothelial cell line (bEnd.3) with Aβ decreased cell viability compared with untreated controls. Aβ also increased permeability to sodium fluorescein, decreased tight junction protein expression, and induced RAGE expression in bEnd.3 cells. Knockdown of RAGE prevented the increased sodium fluorescein permeability and the decreased expression of tight junction proteins. These results indicate that Aβ-RAGE interactions induce endothelial cell defects and may contribute to BBB disruption.

## Interactions of CAP37, neutrophil elastase, and cathepsin G with Aβ and RAGE

Proteins that bind with either RAGE or Aβ would likely have important implications for AD and other neuroinflammatory diseases. Our recent report [10] was the first to demonstrate interactions of CAP37, neutrophil elastase, and cathepsin G with RAGE and also with Aβ_1–42_. Results from ELISAs and far-dot blot assays revealed binding of all three neutrophil proteins to both Aβ_1–42_ and RAGE. Strong binding of CAP37 and cathepsin G to RAGE was observed. Less binding of neutrophil elastase to RAGE was observed. Similarly, a high-binding affinity of Aβ_1–42_ to CAP37 and cathepsin G was measured, whereas binding of Aβ_1–42_ to neutrophil elastase occurred with a much lower affinity.

**Fig. 4:**
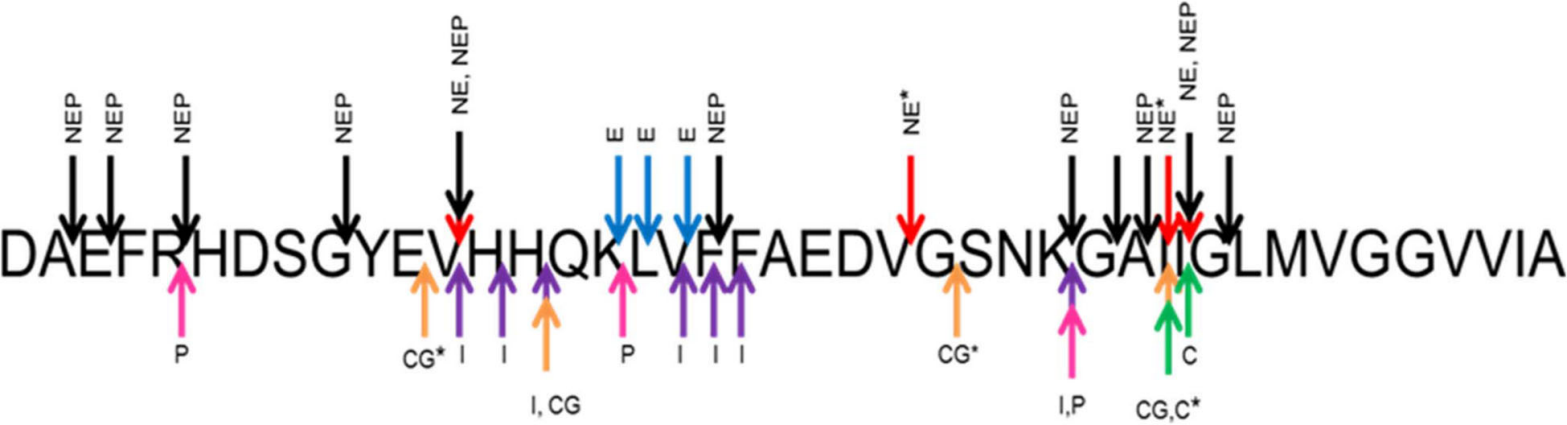
Sites of Aβ_1–42_ cleavage by Aβ degrading proteases. The amino acid sequence of Aβ_1–42_ is shown indicating the sites at which neprilysin (NEP, black arrows), endothelin-converting enzyme (E, blue arrows), neutrophil elastase (NE, red arrows), plasmin (P, pink arrows), cathepsin G (CG, orange arrows), insulin-degrading enzyme (I, purple arrows), and CAP37 (C, green arrows) cleave Aβ_1–42_. Asterisks (*) represent sites that are cleaved only by CAP37, neutrophil elastase, or cathepsin G

Since neutrophil elastase and cathepsin G were known to have proteolytic activity, we investigated whether they had proteolytic activity on Aβ_1–42_. Interestingly, we demonstrated that all three neutrophil proteins, including CAP37, could cleave Aβ_1–42_ using MALDI-TOF mass spectrometry. Neutrophil elastase and cathepsin G cleaved Aβ_1–42_ within seconds, and formed multiple fragment products of Aβ_1–42_ peptide within 1 h of incubation with Aβ_1–42_. CAP37 processed Aβ_1–42_ more slowly, cleaving the majority of full-length Aβ_1–42_ (~ 90%) within 5 h of incubation. Two fragment products of Aβ_1–42_ were formed by CAP37. By performing tandem mass spectrometry, we demonstrated that all three neutrophil proteases cleaved Aβ_1–42_ between residues Ile^31^ and Ile^32^, and CAP37 and neutrophil elastase between residues Ile^32^ and Gly^33^ (Fig. 4). Neutrophil elastase also cleaved Aβ_1–42_ at Val^12^-His^13^ and Val^24^-Gly^25^ (Fig. 4, red arrows). Cathepsin G cleaved Aβ at Glu^11^-Val^12^, His^14^-Gln^15^, and Gly^25^-Ser^26^ (Fig. 4, orange arrows).

Our recent study demonstrated that CAP37 and neutrophil elastase disrupted the binding of Aβ to RAGE. However, CAP37 was not strongly competitive with Aβ for the binding to RAGE, and the cleavage of Aβ by CAP37 occurred at a very slow rate. Therefore, since we observed CAP37 binding to Aβ with a high affinity, we predicted that the mechanism by which CAP37 decreased Aβ binding to RAGE was by CAP37 binding and sequestering Aβ away from RAGE (“quenching” of Aβ). We proposed a different mechanism for neutrophil elastase. Since neutrophil elastase did not bind with a high affinity to Aβ, but rapidly degraded Aβ, we predicted that the mechanism by which neutrophil elastase decreased Aβ binding to RAGE was by degrading Aβ.

## Brain cell and tissue sources of neutrophil granule proteins

It is known that the neutrophil granule proteins CAP37, neutrophil elastase, and cathepsin G are expressed in and secreted from neutrophils as well as some other cells [25, 29, 49, 50]. We have since revealed the expression of these proteins in non-neutrophil cells of the brain **(**Table 2**)**. AZU1 (CAP37) mRNA was observed in brain cells including human primary neurons and microglia. However, the protein was only observed in neurons. CAP37 was upregulated in pyramidal neurons of AD patients. CAP37 may be translated and expressed in glial cells during other diseases or conditions, but this has not been demonstrated to date. In contrast to AZU1 (CAP37), ELANE (neutrophil elastase), and CTSG (cathepsin G) transcripts were not significantly upregulated in patients with AD. Therefore, among the three neutrophil proteins, CAP37 is the only one with expression levels that positively correlated with AD pathology.

**Table 2:** Expression of neutrophil proteins or mRNAs reported in brain cells

	Neurons	Microglia	Astrocytes
CAP37	Yes (9)	Yes (9)	No
Neutrophil elastase	No	Yes (9, 55)	No
Cathepsin G	No	Yes (9, 56)	No

Although ELANE was not upregulated in AD patients, we did observe ELANE mRNA in microglia, confirming findings from other labs that detected neutrophil elastase and cathepsin G proteins in murine microglia [55, 56].

A previous study by Nakajima et al. [55] revealed neutrophil elastase in microglia conditioned medium and demonstrated de novo synthesis of neutrophil elastase in rat microglial cells. Since neutrophil elastase concentration increased in the culture medium over time and decreased in response to LPS, it was believed to be secreted from the microglia. Therefore, neutrophil elastase secreted from microglial cells could directly interact with extracellular Aβ and RAGE on membranes of various brain cells. The recent studies that demonstrated that neutrophils extravasated into the brains of AD mice [24] indicate that neutrophils could be a potential source of increased neutrophil proteins in the brain interstitial fluid during AD. Since CAP37 is expressed within neurons, and neutrophil elastase and cathepsin G have been detected in microglia, these proteins could be released from neurons or microglia.

Necrosis of neurons or microglia could also allow for the release of CAP37, neutrophil elastase, and/or cathepsin G into the extracellular fluid. Aβ, which accumulates intracellularly in neurons [109] has been found to cause deleterious effects on the ubiquitin proteasome system and mitochondria. CAP37 expressed within neurons may also interact with Aβ that is produced by neurons or with Aβ that is taken up by cells from the extracellular space and accumulates intracellularly [109]. Taken together, these findings from our lab and others demonstrate the expression of CAP37, neutrophil elastase, and cathepsin G in non-neutrophil cells within the brain parenchyma. Therefore, CAP37, neutrophil elastase, and cathepsin G may be able to interact with Aβ and RAGE in the brain.

## Potential effects of CAP37, neutrophil elastase, and cathepsin G on Aβ and RAGE

Binding of CAP37, neutrophil elastase, and cathepsin G to Aβ and RAGE [10] could modulate the neuroinflammation and neurotoxicity induced by Aβ-RAGE signaling. If CAP37 and neutrophil elastase decrease Aβ-RAGE signaling on astrocytes and endothelial cells, they may decrease oxidative stress that is induced by Aβ-RAGE on these cells. If this binding and signaling are inhibited on microglia and neurons, the activation of NF-κB in these cells may be prevented, leading to decreased neuroinflammation. As mentioned, RAGE also facilitates the passage of Aβ across the BBB [103, 104]. If the neutrophil proteins were to disrupt the binding of Aβ to RAGE in the periphery, they might help prevent Aβ transport into the brain parenchyma.

To our knowledge, the functions and/or neurotoxic effects of most of the specific fragments of Aβ that were generated by CAP37, neutrophil elastase, and cathepsin G [10] have not been determined. In a study by Hernandez-Guillamon et al., [110] the cytotoxicity of smaller fragments of Aβ peptide on human cerebral microvascular endothelial cells and on SH-SY5Y neuroblastoma cells was assessed. The Aβ peptides Aβ_1–40_, Aβ_1–34_, Aβ_1–30_, and Aβ_1–16_ were all found to be less cytotoxic than Aβ_1–42_. The results from this study support the notion that degradation of Aβ_1–42_ is neuroprotective. In agreement with this notion, other investigators have studied the potential neuroprotective effects of Aβ-degrading enzymes. This research was sparked by a 2002 study by Iwata et al. [111] which demonstrated that neprilysin degraded Aβ, and that inhibition of neprilysin (NEP) resulted in increased Aβ deposition in the brain. Since then, various other enzymes that possess proteolytic activity against Aβ have been identified [112]. Many of these enzymes are being considered as therapeutic targets in AD and other neurodegenerative diseases.

The majority of Aβ-degrading enzymes have been identified within cells of the brain parenchyma, as well as other tissues in the periphery [112–114]. Each of the enzymes differs with respect to their cellular and subcellular localizations, their optimal pH for activity, the sites at which they cleave Aβ, kinetics of Aβ cleavage, and their preferred assembly state of Aβ for cleavage (monomers, oligomers, protofibrils, or fibrils). NEP, insulin-degrading enzyme (IDE), and endothelin-converting enzymes (ECE-1 and -2) are some of the most well-known and characterized Aβ-degrading proteases [112]. To our knowledge, we were the first group to report neutrophil serine proteases degrading Aβ. NEP and IDE cleave Aβ at Val^12^-His^13^ and IDE cleaves at His^14^-Gln^15^ [114], which are sites that are also cleaved by neutrophil elastase and cathepsin G, respectively. Some of the other sites at which CAP37, neutrophil elastase, and cathepsin G cleave Aβ are distinct from other Aβ-degrading enzymes [114] **(**Fig. 4**)**. Thus, neutrophil serine proteases may be able to act synergistically with these other enzymes.

Our in vitro findings indicate that CAP37, neutrophil elastase, and cathepsin G may be able to prevent the activation of RAGE by Aβ [10]. In this way, CAP37, neutrophil elastase, and cathepsin G might act as RAGE inhibitors to prevent RAGE signaling. However, whether each of these proteins would prevent RAGE activation by Aβ in cell culture or in vivo must be investigated. CAP37, neutrophil elastase, and cathepsin G bind to RAGE, but whether these neutrophil proteins would increase or decrease RAGE signaling independently of Aβ is also unknown. We did not observe CAP37 protein in microglial cells [9]. However, the activation of microglial cells in response to CAP37 suggests that there are one or more receptors for CAP37 on microglia. Since RAGE is expressed in microglia, [89] it could be one of these receptors. CAP37 induces some of the same cellular functions induced by other RAGE ligands. Similar to CAP37, the RAGE ligands Aβ and S100B induce the release of pro-inflammatory cytokines from microglia [8, 97, 115, 116]. Like CAP37, other RAGE ligands (S100B, Aβ, HMGB1) induce migration of monocytes and/or microglia [8, 47, 116–120]. CAP37 and AGEs increase the expression of the adhesion proteins ICAM-1 and VCAM-1 on endothelial cells [43, 119]. Our finding that CAP37 binds to RAGE supports the possibility that CAP37 may act as a RAGE agonist. Further studies must be conducted to determine whether CAP37 contributes to microglial activation during AD and whether this activation involves RAGE.

## Conclusions

Recent findings from our lab and others emphasize the importance of delineating the roles of neutrophils and neutrophil proteins in neuroinflammation and AD. Neutrophils that cross into the brain parenchyma of patients with AD may contribute to neuronal damage and cognitive decline. However, specific neutrophil granule proteins may help eliminate pathogenic aggregates, such as Aβ plaques. Therefore, it seems plausible that the neutrophil as a whole may contribute to AD pathology, although specific functions of the neutrophil-derived molecules may promote clearance of Aβ or pathological debris. In addition, neutrophil proteins expressed in other non-neutrophil cells in the brain may be involved in AD. Our observations that CAP37 is expressed in neurons, upregulated in brains of AD patients, and induced by Aβ in neurons and endothelial cells indicate that CAP37 responds to and may influence AD pathology. CAP37, neutrophil elastase, and cathepsin G may all regulate neuroinflammation in AD by binding and cleaving Aβ. The cleavage of Aβ by neutrophil proteins could be important for clearing Aβ in the brain and/or the vasculature. Furthermore, the neutrophil proteins may modulate AD pathology by disrupting the Aβ-RAGE interaction, which is known to contribute to neuroinflammation and oxidative stress. These novel findings suggest a neuroprotective role for CAP37, neutrophil elastase, and cathepsin G. However, the destructive nature of neutrophil elastase and cathepsin G might contribute to neurotoxicity if they are not kept in check by protease inhibitors. Several questions regarding the exact roles of neutrophil granule proteins in AD remain to be resolved. A number of these questions are listed in Table 3**.** Further research should provide knowledge of which functions of neutrophil proteins, including CAP37, neutrophil elastase, and cathepsin G, may contribute to chronic neuroinflammation in AD or other neurodegenerative diseases and whether specific mechanisms may be harnessed for therapeutic development.

**Table 3:** Outstanding questions to be resolved in future studies

1. Do CAP37, neutrophil elastase, and cathepsin G activate RAGE signaling upon binding to RAGE?	
2. Do CAP37, neutrophil elastase, and cathepsin G decrease neuroinflammation by inhibiting Aβ from binding to RAGE?	
3. Could neutrophil granule proteins affect the aggregation of Aβ_1–42_?	
4. Against what specific Aβ_1–42_ aggregates (monomers, oligomers, fibrils) do CAP37, neutrophil elastase, and cathepsin G have proteolytic activity?	
5. Is the density of neutrophil infiltration into the brain during AD sufficient to allow for significant effects of the neutrophil granule proteins? Or would potential effects be primarily due to expression of neutrophil granule proteins in resident brain cells?	
6. Would increasing the expression or activity of CAP37, neutrophil elastase, and cathepsin G in neutrophils, neurons, or glial cells decrease amyloid burden, tau tangle formation, and cognitive deficits associated with AD?	

## Abbreviations

AD: Alzheimer’s disease
AGEs: Advanced glycation end products
APP: Amyloid precursor protein
Aβ: Amyloid beta
Aβ_1–40_: Amyloid beta 1–40
BBB: Blood-brain barrier
bEnd.3: Brain endothelial cell line
CAP37: The cationic antimicrobial protein of m.w. 37 kDa
CCL5: Chemokine ligand 5
CNS: Central nervous system
CXCL2: Chemokine (C-X-C motif) ligand 2
DLBD: Diffuse Lewy body dementia
ECE-1: Endothelin-converting enzyme-1
ECE-2: Endothelin-converting enzyme-2
FTD: Frontotemporal dementia
HMGB-1: High mobility group box 1
ICAM-1: Intercellular adhesion molecule 1
IDE: Insulin-degrading enzyme
IFN-γ: Interferon gamma
IL-1: Interleukin-1
IL-17: Interleukin-17
IL-1β: Interleukin-1 beta
IL-6: Interleukin-6
LFA-1: Lymphocyte function-associated antigen-1
LPS: Lipopolysaccharide
LRP1: Low density lipoprotein receptor-related protein 1
mAPP: Mutant amyloid precursor protein
M-CSF: Macrophage colony stimulating factor
MIP-1α: Macrophage inflammatory protein 1-α
MMP-9: Matrix metalloproteinase-9
NEP: Neprilysin
NF-κB: Nuclear factor kappa-light-chain-enhancer of activated B cells
PARs: Protease-activated receptors
PECAM-1: Platelet endothelial cell adhesion molecule 1
PRR: Pattern recognition receptor
qRT-PCR: Quantitative reverse transcriptase polymerase chain reaction
RAGE: The receptor for advanced glycation end products
SDF-1: Stromal cell-derived factor 1
sRAGE: Soluble RAGE
Tg: Transgenic
TNF-α: Tumor necrosis factor alpha
VaD: Vascular dementia
VCAM-1: Vascular cell adhesion protein 1

## References

[1] Mocsai A (2013). Diverse novel functions of neutrophils in immunity, inflammation, and beyond. J Exp Med.

[2] Kruger P, Saffarzadeh M, Weber ANR, Rieber N, Radsak M, von Bernuth H, Benarafa C, Roos D, Skokowa J, Hartl D (2015). Neutrophils: between host defence, immune modulation, and tissue injury. PLoS Pathog.

[3] Lieber JG, Webb S, Suratt BT, Young SK, Johnson GL, Keller GM, Worthen GS (2004). The in vitro production and characterization of neutrophils from embryonic stem cells. Blood.

[4] Gifford AM, Chalmers JD (2014). The role of neutrophils in cystic fibrosis. Curr Opin Hematol.

[5] Williams TJ, Jose PJ (2001). Neutrophils in chronic obstructive pulmonary disease. Novartis Found Symp.

[6] Wright HL, Moots RJ, Edwards SW (2014). The multifactorial role of neutrophils in rheumatoid arthritis. Nat Rev Rheumatol.

[7] Pereira HA, Kumar P, Grammas P (1996). Expression of CAP37, a novel inflammatory mediator, in Alzheimer's disease. Neurobiol Aging.

[8] Pereira HA, Ruan X, Kumar P (2003). Activation of microglia: a neuroinflammatory role for CAP37. Glia.

[9] Brock AJ, Kasus-Jacobi A, Lerner M, Logan S, Adesina AM, Anne Pereira H (2015). The antimicrobial protein, CAP37, is upregulated in pyramidal neurons during Alzheimer's disease. Histochem Cell Biol.

[10] Stock AJ, Kasus-Jacobi A, Wren JD, Sjoelund VH, Prestwich GD, Pereira HA (2016). The role of neutrophil proteins on the amyloid Beta-RAGE Axis. PLoS One.

[11] Alzheimer’s A (2016). 2016 Alzheimer's disease facts and figures. Alzheimers Dement.

[12] Querfurth HW, LaFerla FM (2010). Alzheimer's disease. N Engl J Med.

[13] Gong CX, Liu F, Iqbal K. Multifactorial hypothesis and multi-targets for Alzheimer's disease. J Alzheimers Dis. 2016; 10.3233/JAD-179921. Epub ahead of print 10.3233/JAD-179921 29562523

[14] Malm T, Koistinaho M, Muona A, Magga J, Koistinaho J (2010). The role and therapeutic potential of monocytic cells in Alzheimer's disease. Glia.

[15] Togo T, Akiyama H, Iseki E, Kondo H, Ikeda K, Kato M, Oda T, Tsuchiya K, Kosaka K (2002). Occurrence of T cells in the brain of Alzheimer's disease and other neurological diseases. J Neuroimmunol.

[16] Giri R, Shen Y, Stins M, Du Yan S, Scmidt AM, Stern D, Kim KS, Zlokovic B, Kalra VK (2000). Beta-amyloid-induced migration of monocytes across human brain endothelial cells invovles RAGE and PECAM-1. Am J Physiol Cell Physiol.

[17] Fiala M, Zhang L, Gan X, Sherry B, Taub D, Graves MC, Hama S, Way D, Weinand M, Witte M, Lorton D, Kuo YM, Roher AE (1998). Amyloid-beta induces chemokine secretion and monocyte migration across a human blood--brain barrier model. Mol Med.

[18] Hohsfield LA, Humpel C (2015). Migration of blood cells to β-amyloid plaques in Alzheimer's disease. Exp Gerontol.

[19] Town T, Tan J, Flavell RA, Mullan M (2005). T-cells in Alzheimer's disease. NeuroMolecular Med.

[20] Buckwalter MS, Coleman BS, Buttini M, Barbour R, Schenk D, Games D, Seubert P, Wyss-Coray T (2006). Increased T cell recruitment to the CNS after amyloid beta 1-42 immunization in Alzheimer's mice overproducing transforming growth factor-beta 1. J Neurosci.

[21] Scali C, Prosperi C, Bracco L, Piccini C, Baronti R, Ginestroni A, Sorbi S, Pepeu G, Casamenti F (2002). Neutrophils CD11b and fibroblasts PGE(2) are elevated in Alzheimer's disease. Neurobiol Aging.

[22] Vitte J, Michel BF, Bongrand P, Gastaut JL (2004). Oxidative stress level in circulating neutrophils is linked to neurodegenerative diseases. J Clin Immunol.

[23] Baik SH, Cha MY, Hyun YM, Cho H, Hamza B, Kim DK, Han SH, Choi H, Kim KH, Moon M (2014). Migration of neutrophils targeting amyloid plaques in Alzheimer's disease mouse model. Neurobiol Aging.

[24] Zenaro E, Pietronigro E, Della Bianca V, Piacentino G, Marongiu L, Budui S, Turano E, Rossi B, Angiari S, Dusi S (2015). Neutrophils promote Alzheimer's disease-like pathology and cognitive decline via LFA-1 integrin. Nat Med.

[25] Pham CT (2006). Neutrophil serine proteases: specific regulators of inflammation. Nat Rev Immunol.

[26] Gullberg U, Andersson E, Garwicz D, Lindmark A, Olsson I (1997). Biosynthesis, processing and sorting of neutrophil proteins: insight into neutrophil granule development. Eur J Haematol.

[27] Borregaard N, Cowland JB (1997). Granules of the human neutrophilic polymorphonuclear leukocyte. Blood.

[28] Borregaard N, Sorensen OE, Theilgaard-Monch K (2007). Neutrophil granules: a library of innate immunity proteins. Trends Immunol.

[29] Soehnlein O, Lindbom L (2009). Neutrophil-derived azurocidin alarms the immune system. J Leukoc Biol.

[30] Tapper H, Karlsson A, Morgelin M, Flodgaard H, Herwald H (2002). Secretion of heparin-binding protein from human neutrophils is determined by its localization in azurophilic granules and secretory vesicles. Blood.

[31] Pereira HA, Spitznagel JK, Pohl J, Wilson DE, Morgan J, Palings I, Larrick JW (1990). CAP 37, a 37 kD human neutrophil granule cationic protein shares homology with inflammatory proteinases. Life Sci.

[32] Karlsen S, Iversen LF, Larsen IK, Flodgaard HJ, Kastrup JS (1998). Atomic resolution structure of human HBP/CAP37/azurocidin. Acta Crystallogr D Biol Crystallogr.

[33] Korkmaz B, Horwitz MS, Jenne DE, Gauthier F (2010). Neutrophil elastase, proteinase 3, and cathepsin G as therapeutic targets in human diseases. Pharmacol Rev.

[34] Lindmark A, Garwicz D, Rasmussen PB, Flodgaard H, Gullberg U (1999). Characterization of the biosynthesis, processing, and sorting of human HBP/CAP37/azurocidin. J Leukoc Biol.

[35] Owen CA, Campbell EJ (1999). The cell biology of leukocyte-mediated proteolysis. J Leukoc Biol.

[36] Morgan JG, Pereira HA, Sukiennicki T, Spitznagel JK, Larrick JW (1991). Human neutrophil granule cationic protein CAP37 is a specific macrophage chemotaxin that shares homology with inflammatory proteinases. Adv Exp Med Biol.

[37] Wang J, Shafqat J, Hall K, Stahlberg M, Wivall-Helleryd IL, Bouzakri K, Zierath JR, Brismar K, Jornvall H, Lewitt MS (2006). Specific cleavage of insulin-like growth factor-binding protein-1 by a novel protease activity. Cell Mol Life Sci.

[38] Brandt K, Lundell K, Brismar K (2011). Neutrophil-derived azurocidin cleaves insulin-like growth factor-binding protein-1, -2 and -4. Growth Hormon IGF Res.

[39] Pereira HA (1995). CAP37, a neutrophil-derived multifunctional inflammatory mediator. J Leukoc Biol.

[40] Soehnlein O, Xie X, Ulbrich H, Kenne E, Rotzius P, Flodgaard H, Eriksson EE, Lindbom L (2005). Neutrophil-derived heparin-binding protein (HBP/CAP37) deposited on endothelium enhances monocyte arrest under flow conditions. J Immunol.

[41] Heinzelmann M, Mercer-Jones MA, Flodgaard H, Miller FN (1998). Heparin-binding protein (CAP37) is internalized in monocytes and increases LPS-induced monocyte activation. J Immunol.

[42] Pereira HA (2001). Cationic antimicrobial protein of Mr 37 kDa: a multifunctional inflammatory protein. Chin Med J.

[43] Lee TD, Gonzalez ML, Kumar P, Grammas P, Pereira HA (2003). CAP37, a neutrophil-derived inflammatory mediator, augments leukocyte adhesion to endothelial monolayers. Microvasc Res.

[44] Rasmussen PB, Bjorn S, Hastrup S, Nielsen PF, Norris K, Thim L, Wiberg FC, Flodgaard H (1996). Characterization of recombinant human HBP/CAP37/azurocidin, a pleiotropic mediator of inflammation-enhancing LPS-induced cytokine release from monocytes. FEBS Lett.

[45] Gautam N, Olofsson AM, Herwald H, Iversen LF, Lundgren-Akerlund E, Hedqvist P, Arfors KE, Flodgaard H, Lindbom L (2001). Heparin-binding protein (HBP/CAP37): a missing link in neutrophil-evoked alteration of vascular permeability. Nat Med.

[46] Gonzalez ML, Ruan X, Kumar P, Grammas P, Pereira HA (2004). Functional modulation of smooth muscle cells by the inflammatory mediator CAP37. Microvasc Res.

[47] Pereira HA, Shafer WM, Pohl J, Martin LE, Spitznagel JK (1990). CAP37, a human neutrophil-derived chemotactic factor with monocyte specific activity. J Clin Invest.

[48] Soehnlein O, Kai-Larsen Y, Frithiof R, Sorensen OE, Kenne E, Scharffetter-Kochanek K, Eriksson EE, Herwald H, Agerberth B, Lindbom L (2008). Neutrophil primary granule proteins HBP and HNP1-3 boost bacterial phagocytosis by human and murine macrophages. J Clin Invest.

[49] Lee TD, Gonzalez ML, Kumar P, Chary-Reddy S, Grammas P, Pereira HA (2002). CAP37, a novel inflammatory mediator. Am J Pathol.

[50] Ruan X, Chodosh J, Callegan MC, Booth MC, Lee TD, Kumar P, Gilmore MS, Pereira HA (2002). Corneal expression of the inflammatory mediator CAP37. Invest Ophthalmol Vis Sci.

[51] Linder A, Christensson B, Herwald H, Bjorck L, Akesson P (2009). Heparin-binding protein: an early marker of circulatory failure in sepsis. Clin Infect Dis.

[52] Linder A, Akesson P, Brink M, Studahl M, Bjorck L, Christensson B (2011). Heparin-binding protein: a diagnostic marker of acute bacterial meningitis. Crit Care Med.

[53] Takasaki J, Ogawa Y (1997). Granulocyte elastase activity measurement in the cerebrospinal fluid of patients with purulent meningitis. Acta Paediatr Jpn.

[54] Semple BD, Trivedi A, Gimlin K, Noble-Haeusslein LJ (2015). Neutrophil elastase mediates acute pathogenesis and is a determinant of long-term behavioral recovery after traumatic injury to the immature brain. Neurobiol Dis.

[55] Nakajima K, Shimojo M, Hamanoue M, Ishiura S, Sugita H, Kohsaka S (1992). Identification of elastase as a secretory protease from cultured rat microglia. J Neurochem.

[56] Burster T, Beck A, Poeschel S, Oren A, Baechle D, Reich M, Roetzschke O, Falk K, Boehm BO, Youssef S (2007). Interferon-gamma regulates cathepsin G activity in microglia-derived lysosomes and controls the proteolytic processing of myelin basic protein in vitro. Immunology.

[57] Saadoun S, Waters P, MacDonald C, Bell BA, Vincent A, Verkman AS, Papadopoulos MC (2012). Neutrophil protease inhibition reduces neuromyelitis optica-immunoglobulin G-induced damage in mouse brain. Ann Neurol.

[58] Faraday N, Schunke K, Saleem S, Fu J, Wang B, Zhang J, Morrell C, Dore S (2013). Cathepsin G-dependent modulation of platelet thrombus formation in vivo by blood neutrophils. PLoS One.

[59] Xavier AL, Menezes JR, Goldman SA, Nedergaard M (2014). Fine-tuning the central nervous system: microglial modelling of cells and synapses. Philos Trans R Soc Lond Ser B Biol Sci.

[60] von Bernhardi R, Eugenin-von Bernhardi L, Eugenin J (2015). Microglial cell dysregulation in brain aging and neurodegeneration. Front Aging Neurosci.

[61] Fu R, Shen Q, Xu P, Luo JJ, Tang Y (2014). Phagocytosis of microglia in the central nervous system diseases. Mol Neurobiol.

[62] Kettenmann H, Hanisch UK, Noda M, Verkhratsky A (2011). Physiology of microglia. Physiol Rev.

[63] Kettenmann H, Kirchhoff F, Verkhratsky A (2013). Microglia: new roles for the synaptic stripper. Neuron.

[64] Dou Y, Wu HJ, Li HQ, Qin S, Wang YE, Li J, Lou HF, Chen Z, Li XM, Luo QM, Duan S (2012). Microglial migration mediated by ATP-induced ATP release from lysosomes. Cell Res.

[65] Lull ME, Block ML (2010). Microglial activation and chronic neurodegeneration. Neurotherapeutics.

[66] Heneka MT, Carson MJ, El Khoury J, Landreth GE, Brosseron F, Feinstein DL, Jacobs AH, Wyss-Coray T, Vitorica J, Ransohoff RM (2015). Neuroinflammation in Alzheimer's disease. Lancet Neurol.

[67] Edison P, Archer HA, Gerhard A, Hinz R, Pavese N, Turkheimer FE, Hammers A, Tai YF, Fox N, Kennedy A (2008). Microglia, amyloid, and cognition in Alzheimer's disease: an [11C](R)PK11195-PET and [11C]PIB-PET study. Neurobiol Dis.

[68] Gerhard A, Pavese N, Hotton G, Turkheimer F, Es M, Hammers A, Eggert K, Oertel W, Banati RB, Brooks DJ (2006). In vivo imaging of microglial activation with [11C](R)-PK11195 PET in idiopathic Parkinson's disease. Neurobiol Dis.

[69] Akiyama H, Barger S, Barnum S, Bradt B, Bauer J, Cole GM, Cooper NR, Eikelenboom P, Emmerling M, Fiebich BL (2000). Inflammation and Alzheimer's disease. Neurobiol Aging.

[70] Wang Q, Liu Y, Zhou J (2015). Neuroinflammation in Parkinson's disease and its potential as therapeutic target. Transl Neurodegener.

[71] Kleinberger G, Yamanishi Y, Suarez-Calvet M, Czirr E, Lohmann E, Cuyvers E, Struyfs H, Pettkus N, Wenninger-Weinzierl A, Mazaheri F (2014). TREM2 mutations implicated in neurodegeneration impair cell surface transport and phagocytosis. Sci Transl Med.

[72] Krabbe G, Halle A, Matyash V, Rinnenthal JL, Eom GD, Bernhardt U, Miller KR, Prokop S, Kettenmann H, Heppner FL (2013). Functional impairment of microglia coincides with beta-amyloid deposition in mice with Alzheimer-like pathology. PLoS One.

[73] Serrano-Pozo A, Frosch MP, Masliah E, Hyman BT (2011). Neuropathological alterations in Alzheimer disease. Cold Spring Harb Perspect Med.

[74] Pahlman LI, Morgelin M, Eckert J, Johansson L, Russell W, Riesbeck K, Soehnlein O, Lindbom L, Norrby-Teglund A, Schumann RR (2006). Streptococcal M protein: a multipotent and powerful inducer of inflammation. J Immunol.

[75] Zen K, Guo YL, Li LM, Bian Z, Zhang CY, Liu Y (2011). Cleavage of the CD11b extracellular domain by the leukocyte serprocidins is critical for neutrophil detachment during chemotaxis. Blood.

[76] Griffith GL, Russell RA, Kasus-Jacobi A, Thavathiru E, Gonzalez ML, Logan S, Pereira HA (2013). CAP37 activation of PKC promotes human corneal epithelial cell chemotaxis. Invest Ophthalmol Vis Sci.

[77] Chertov O, Ueda H, Xu LL, Tani K, Murphy WJ, Wang JM, Howard OM, Sayers TJ, Oppenheim JJ (1997). Identification of human neutrophil-derived cathepsin G and azurocidin/CAP37 as chemoattractants for mononuclear cells and neutrophils. J Exp Med.

[78] Sun R, Iribarren P, Zhang N, Zhou Y, Gong W, Cho EH, Lockett S, Chertov O, Bednar F, Rogers TJ (2004). Identification of neutrophil granule protein cathepsin G as a novel chemotactic agonist for the G protein-coupled formyl peptide receptor. J Immunol.

[79] Woloszynek JC, Hu Y, Pham CT (2012). Cathepsin G-regulated release of formyl peptide receptor agonists modulate neutrophil effector functions. J Biol Chem.

[80] Alberelli MA, De Candia E (2014). Functional role of protease activated receptors in vascular biology. Vasc Pharmacol.

[81] Sambrano GR, Huang W, Faruqi T, Mahrus S, Craik C, Coughlin SR (2000). Cathepsin G activates protease-activated receptor-4 in human platelets. J Biol Chem.

[82] Renesto P, Si-Tahar M, Moniatte M, Balloy V, Van Dorsselaer A, Pidard D, Chignard M (1997). Specific inhibition of thrombin-induced cell activation by the neutrophil proteinases elastase, cathepsin G, and proteinase 3: evidence for distinct cleavage sites within the aminoterminal domain of the thrombin receptor. Blood.

[83] Bierhaus A, Humpert PM, Morcos M, Wendt T, Chavakis T, Arnold B, Stern DM, Nawroth PP (2005). Understanding RAGE, the receptor for advanced glycation end products. J Mol Med (Berl).

[84] Fritz G (2011). RAGE: a single receptor fits multiple ligands. Trends Biochem Sci.

[85] Lue LF, Walker DG, Brachova L, Beach TG, Rogers J, Schmidt AM, Stern DM, Yan SD (2001). Involvement of microglial receptor for advanced glycation end products (RAGE) in Alzheimer's disease: identification of a cellular activation mechanism. Exp Neurol.

[86] Villarreal A, Seoane R, Gonzalez Torres A, Rosciszewski G, Angelo MF, Rossi A, Barker PA, Ramos AJ (2014). S100B protein activates a RAGE-dependent autocrine loop in astrocytes: implications for its role in the propagation of reactive gliosis. J Neurochem.

[87] Kook SY, Hong HS, Moon M, Ha CM, Chang S, Mook-Jung I (2012). Abeta(1)(−)(4)(2)-RAGE interaction disrupts tight junctions of the blood-brain barrier via ca(2)(+)-calcineurin signaling. J Neurosci.

[88] Kierdorf K, Fritz G (2013). RAGE regulation and signaling in inflammation and beyond. J Leukoc Biol.

[89] Yan SD, Chen X, Fu J, Chen M, Zhu H, Roher A, Slattery T, Zhao L, Nagashima M, Morser J (1996). RAGE and amyloid-beta peptide neurotoxicity in Alzheimer's disease. Nature.

[90] Chuah YK, Basir R, Talib H, Tie TH, Nordin N (2013). Receptor for advanced glycation end products and its involvement in inflammatory diseases. Int J Inflamm.

[91] Murphy MP, LeVine H (2010). Alzheimer's disease and the amyloid-beta peptide. J Alzheimers Dis.

[92] Borchelt DR, Thinkakaran G, Eckman CB, Lee MK, Davenport F, Ratovitsky T, Prada CM, Kim G, Seekins S, Yager D, Slunt HH, Wang R, Seeger M, Al L, Gandy SE, Copeland NG, Jenkins NA, Price DL, Younkin SG, Sisodia SS (1996). Familial Alzheimer's disease-linked presenilin 1 variants elevate Abeta1-42/1-40 ratio in vitro and in vivo. Neuron.

[93] Eckman CB, Mehta ND, Crook R, Perez-tur J, Prihar G, Pfeiffer E, Graff-Radford N, Hinder P, Yager D, Zenk B, Refolo LM, Prada CM, Younkin SG, Hutton M, Hardy J (1997). A new pathogenic mutation in the APP gene (I716V) increases the relative proportion of A beta 42(43). Hum Mol Genet.

[94] Mayeux R, Tang MX, Jacobs DM, Manly J, Bell K, Merchant C, Small SA, Stern Y, Wisniewski HM, Mehta PD (1999). Plasma amyloid beta-peptide and incipient Alzheimer's disease. Ann Neurol.

[95] Hardy JA, Higgins GA (1992). Alzheimer's disease: the amyloid cascade hypothesis. Science.

[96] Liu R, Wu CX, Zhou D, Yang F, Tian S, Zhang L, Zhang TT, Du GH (2012). Pinocembrin protects against beta-amyloid-induced toxicity in neurons through inhibiting receptor for advanced glycation end products (RAGE)-independent signaling pathways and regulating mitochondrion-mediated apoptosis. BMC Med.

[97] Fang F, Lue LF, Yan S, Xu H, Luddy JS, Chen D, Walker DG, Stern DM, Yan S, Schmidt AM (2010). RAGE-dependent signaling in microglia contributes to neuroinflammation, Abeta accumulation, and impaired learning/memory in a mouse model of Alzheimer's disease. FASEB J.

[98] Cai Z, Liu N, Wang C, Qin B, Zhou Y, Xiao M, Chang L, Yan LJ, Zhao B (2016). Role of RAGE in Alzheimer's disease. Cell Mol Neurobiol.

[99] Askarova S, Yang X, Sheng W, Sun GY, Lee JC (2011). Role of Abeta-receptor for advanced glycation endproducts interaction in oxidative stress and cytosolic phospholipase A(2) activation in astrocytes and cerebral endothelial cells. Neuroscience.

[100] Carrano A, Hoozemans JJ, van der Vies SM, Rozemuller AJ, van Horssen J, de Vries HE (2011). Amyloid beta induces oxidative stress-mediated blood-brain barrier changes in capillary amyloid angiopathy. Antioxid Redox Signal.

[101] Du Yan S, Zhu H, Fu J, Yan SF, Roher A, Tourtellotte WW, Rajavashisth T, Chen X, Godman GC, Stern D, Schmidt AM (1997). Amyloid-beta peptide-receptor for advanced glycation endproduct interaction elicits neuronal expression of macrophage-colony stimulating factor: a proinflammatory pathway in Alzheimer disease. Proc Natl Acad Sci U S A.

[102] Arancio O, Zhang HP, Chen X, Lin C, Trinchese F, Puzzo D, Liu S, Hegde A, Yan SF, Stern A (2004). RAGE potentiates Abeta-induced perturbation of neuronal function in transgenic mice. EMBO J.

[103] Zhao Z, Nelson AR, Betsholtz C, Zlokovic BV (2015). Establishment and dysfunction of the blood-brain barrier. Cell.

[104] Marques F, Sousa JC, Sousa N, Palha JA (2013). Blood-brain-barriers in aging and in Alzheimer's disease. Mol Neurodegener.

[105] Farkas IG, Czigner A, Farkas E, Dobo E, Soos K, Penke B, Endresz V, Mihaly A (2003). Beta-amyloid peptide-induced blood-brain barrier disruption facilitates T-cell entry into the rat brain. Acta Histochem.

[106] Kook SY, Seok Hong H, Moon M, Mook-Jung I (2013). Disruption of blood-brain barrier in Alzheimer disease pathogenesis. Tissue Barriers.

[107] Wan W, Cao L, Liu L, Zhang C, Kalionis B, Tai X, Li Y, Xia S (2015). Abeta(1-42) oligomer-induced leakage in an in vitro blood-brain barrier model is associated with up-regulation of RAGE and metalloproteinases, and down-regulation of tight junction scaffold proteins. J Neurochem.

[108] Ballabh P, Braun A, Nedergaard M (2004). The blood-brain barrier: an overview: structure, regulation, and clinical implications. Neurobiol Dis.

[109] Kam TI, Gwon Y, Jung YK (2014). Amyloid beta receptors responsible for neurotoxicity and cellular defects in Alzheimer's disease. Cell Mol Life Sci.

[110] Hernandez-Guillamon M, Mawhirt S, Blais S, Montaner J, Neubert TA, Rostagno A, Ghiso J (2015). Sequential amyloid-beta degradation by the matrix metalloproteases MMP-2 and MMP-9. J Biol Chem.

[111] Iwata N, Tsubuki S, Takaki Y, Watanabe K, Sekiguchi M (2000). Identification of the major A beta 1-42-degrading catabolic pathway in brain parenchyma: suppression leads to biochemical and pathological deposition. Nat Med.

[112] Saido T, Leissring MA (2012). Proteolytic degradation of amyloid beta-protein. Cold Spring Harb Perspect Med.

[113] Turner AJ, Fisk L, Nalivaeva NN (2004). Targeting amyloid-degrading enzymes as therapeutic strategies in neurodegeneration. Ann N Y Acad Sci.

[114] Wang DS, Dickson DW, Malter JS (2006). Beta-amyloid degradation and Alzheimer's disease. J Biomed Biotechnol.

[115] Yates SL, Burgess LH, Kocsis-Angle J, Antal JM, Dority MD, Embury PB, Piotrkowski AM, Brunden KR (2000). Amyloid beta and amylin fibrils induce increases in proinflammatory cytokine and chemokine production by THP-1 cells and murine microglia. J Neurochem.

[116] Bianchi R, Kastrisianaki E, Giambanco I, Donato R (2011). S100B protein stimulates microglia migration via RAGE-dependent up-regulation of chemokine expression and release. J Biol Chem.

[117] Fiala M, Zhang L, Gan X, Sherry B, Taub D, Graves MC, Hama S, Way D, Weinand M, Witte M (1998). Amyloid-beta induces chemokine secretion and monocyte migration across a human blood--brain barrier model. Mol Med.

[118] Giri R, Shen Y, Stins M, Du Yan S, Schmidt AM, Stern D, Kim KS, Zlokovic B, Kalra VK (2000). Beta-amyloid-induced migration of monocytes across human brain endothelial cells involves RAGE and PECAM-1. Am J Physiol Cell Physiol.

[119] Kunt T, Forst T, Harzer O, Buchert G, Pfutzner A, Lobig M, Zschabitz A, Stofft E, Engelbach M, Beyer J (1998). The influence of advanced glycation endproducts (AGE) on the expression of human endothelial adhesion molecules. Exp Clin Endocrinol Diabetes.

[120] Rouhiainen A, Kuja-Panula J, Wilkman E, Pakkanen J, Stenfors J, Tuominen RK, Lepantalo M, Carpen O, Parkkinen J, Rauvala H (2004). Regulation of monocyte migration by amphoterin (HMGB1). Blood.

